# The relationship between clinical dishonesty and perceived clinical stress among nursing students in southeast of Iran

**DOI:** 10.1186/s12912-020-00434-w

**Published:** 2020-05-14

**Authors:** Foozieh Rafati, Behnaz Bagherian, Parvin Mangolian shahrbabaki, Zahra Imani Goghary

**Affiliations:** 1 Nursing and midwifery school, Jiroft University of medical sciences, Jiroft, Iran; 2 Nursing Research Center, Razi Faculty of Nursing and Midwifery, Kerman University of Medical Sciences, Kerman, Iran; 3 Department of Nursing and midwifery, Sirjan school of Medical Sciences, Sirjan, Iran

## Abstract

**Background:**

Clinical dishonesty is a complex problem that threatens the health and safety of patients. This study aimed to investigate the relationship between clinical dishonesty and perceived clinical stress in nursing students.

**Method:**

This cross-sectional correlational study was conducted on 395 nursing students from 4 nursing colleges. The data were collected using a demographic information questionnaire, Nursing Student^’^s Perception of Clinical Stressors, and a 12-item researcher-made questionnaire to evaluate the frequency of clinical dishonesty in the previous semester, the frequency of witnessing dishonest behavior among peers, and the perceived severity of unethical behavior.

**Results:**

In this study, 89.1% of the students stated that they had committed at least one dishonest clinical behavior in the previous semester. The frequency of clinical dishonesty was significantly correlated with the frequency of observing dishonesty among peers (*r* = 0.053, *p*<0.01), perceived severity of unethical behavior (*r* = − 0.4, *p*<0.01), and perceived stress of students in the clinical setting (*r* = 0.28, *p*<0.01). Moreover, there were significant differences in the frequency of clinical dishonesty by gender (*p* = 0.006), the interest in the field of study (*p* = 0.004), and academic year (*p* = 0.002).

**Conclusion:**

The frequency of clinical dishonesty among nursing students is high and needs attention. Furthermore, considering the positive relationship between dishonesty and perceived clinical stress, it is essential to teach effective strategies to nursing students to empower them to cope with clinical stress.

## Background

Academic dishonesty is deliberate complicity in the deceptive performance of one’s own or another’s academic work [1], which is an extremely complex issue [2]. According to educational theories, poor learning environments, lack of ethics training by the university [3], poor communication with peers and teachers (according to social psychologists) [4], and some cultural factors are associated with academic dishonesty [5]. Although it is assumed that academic dishonesty among nursing students is lower than that among students of other majors, studies have shown that the trend of dishonest behaviors in nursing students is similar to that of other majors [6]. The continuing growth of academic dishonesty in nursing education is of concern to nursing institutions and educators in many countries [7, 8]. Previous studies have shown that nursing students are likely to commit dishonest behavior in both the clinical setting and the classroom [9, 10]. Some studies showed that 13% of students recorded the vital signs that they had not measured and 2% recorded medications that they had not given to the patients [9, 11]. Another study revealed that 45% of Iranian nursing students have experienced cheating [12]. Park et al. found that 66% of nursing students engaged in unethical clinical behaviors [13]. The results of these studies have raised serious concern among educators and healthcare managers, as research on this topic has been increased in the last years [14].

The reasons reported for unethical behaviors committed by nursing students in clinical practice include overwork, the inappropriate clinical culture of nurses, poor role models, patients’ non-cooperation with students, fear of being rejected by the preceptor or hospital, lack of awareness, high-volume and difficult courses, fear of failure, stress, and pressure for success and competition with peers [12, 13, 15].

Most nursing studies have described academic dishonesty as an ethical or moral problem [2], but academic dishonesty may occur in stressful environments [1]. The stress from teamwork and task work is also an effective factor in committing dishonesty [16]. Clinical education is at the heart of nursing education and is, unfortunately, an important source of stress for nursing students around the world [17, 18]. Therefore, clinical stress as a risk factor for students makes them increasingly vulnerable to dishonest clinical behaviors. On the other hand, for effective and reliable clinical education, having a clear understanding of dishonest and unethical clinical behaviors of nursing students is of high importance [13]. Dishonesty is considered a major threat to learning [12], it hurts patient care and safety [10], and can be a recurring pattern in future professional behavior that puts patients at risk. Therefore, ignoring the clinical dishonesty of nursing students can be problematic, as studies are very limited in this area [14]. This study aimed to investigate the frequency of dishonest clinical behaviors and their relationship with perceived stress in nursing students.

## Methods

This was a cross-sectional correlational study. The research population consisted of students of nursing colleges in 2 southern provinces of Iran (Kerman and Sistan & Baluchistan). Two nursing colleges were randomly selected from each province (4 colleges in total). Then, the first- to fourth-year nursing students were enrolled in the study (395 students). A census method was used for sampling. The inclusion criteria were passing at least one clinical course and the willingness to participate in the study. For data collection, the data collector referred to the instructors by presenting an introduction letter from the head of the school. After obtaining permission from the instructors at the end of the class, he distributed the questionnaires and collected them at the same session (after 15 min). He introduced himself and gave a brief explanation about the purpose of the study, anonymity, data confidentiality, and voluntary participation in the study to students before distributing the questionnaires.

### Data collection tools

A demographic questionnaire, Clinical Dishonesty Questionnaire, and Nursing Student’s Clinical Perceived Stress Scale were used for data collection.

### Demographic questionaire

The participants’ demographic data included age, sex, marital status, school year, student’s clinical work experience, interest in nursing, and the grade point average (GPA) of the previous semester.

### Clinical dishonesty questionnaire

This was a researcher-made questionnaire with 12 items developed based on a review of the literature [10, 13]. Each item contained 3 parts. The first part measured the frequency of doing dishonest behavior in the previous semester (never, once, twice, or more). The second part measured the frequency of witnessing clinical dishonest behaviors committed by classmates in the previous semester (never, once, twice, or more). The scorings of these 2 parts ranged from 0 to 2, respectively. The third section measured the perceived severity of the unethical behavior on a scale ranging from 1 to 4 (unimportant, least important, important, and most important). The mean total score of each part was calculated as the sum of the responses to the items divided by the number of items. The validity of this questionnaire was confirmed by 10 nursing faculty members. Also, its reliability was determined by Cronbach’s alpha for the frequency of doing the dishonest behavior, frequency of witnessing dishonest behavior committed by classmates, and perceived severity of unethical behavior using the archived data of 30 last-year students. The obtained values were 0.79, 0.83, and 0.83, respectively.

### Nursing student^’^s clinical perceived stress scale

This questionnaire was designed by the first author of another study that has not been published yet. It was developed by interviewing first- to fourth-year nursing students, and all its psychometric properties (face validity, content validity, exploratory and confirmatory factor analysis) were confirmed. The questionnaire contains 32 items that are scored based on a 5-point Likert scale, a higher score indicating more clinical stress. The internal consistency of the questionnaire was 0.9.

### Data analysis

The data were analyzed using SPSS (version 16). Data normality was assessed by the Shapiro-Wilk test, which showed that data distribution was not normal. Thus, the Box-Cox transform was used which did not normalize the data. Non-parametric tests were used for data analysis. Mann-Whitney U-test was run to determine the relationship between dishonest clinical behaviors and bivariate demographic variables. Further, Kruskal–Wallis test was used to determine the relationship between dishonest clinical behaviors and multivariate demographic variables (If the test result was significant, the follow-up test with Bonferroni correction was used to examine the intergroup differences). Moreover, Spearman correlation coefficient was employed to find out if the frequency of doing dishonest clinical behaviors was correlated with witnessing such behaviors, perceived severity of dishonest behaviors as unethical behaviors, the stress experienced by the nursing students, and the students’ GPA of the previous semester.

After obtaining approval from the ethics committee of the university and the permission of the university authorities, anonymous questionnaires were distributed among the participants. The questionnaires were collected after being filled out by the students.

The data were collected from November 2018 to January 2019. The questionnaires were collected by a trained MSc nursing student who studied nursing at a different university.

### Ethical considerations

Ethical approval for this study was obtained from “Ethics Committee in Biomedical Research of Jiroft University of Medical Sciences (IR.JMU.REC.1397.31)”. The questionnaires were anonymous. Besides, the confidentiality of the participants’ information and the right to withdraw from the study were explained to them. Moreover, a written consent form was obtained from all participants. The students were asked to submit the signed form and complete the questionnaire if they agreed to participate in the study, or to submit the blank form and questionnaire if they did not wish to participate in the study. Despite the voluntary participation in the study, no student refused to participate in the study.

## Results

The mean age of the participants was 21.85 ± 2.32 years. About half of the participants (201 students) (50.1%) were female and 337 (85.3%) were single. The mean GPA of the students was 16.5 ± 1.4. A total of 182 students (46.1%) were interested in their field of study, 33 (8.1%) were uninterested, and 177 (44.8%) were somewhat interested.

### Frequency of dishonest behaviors and their perceived severity as being unethical

Out of 395 students, 352 (89.1%) had engaged in one or more dishonest clinical behavior in the previous semester. Moreover, 379 (95.9%) had witnessed at least one dishonest behavior by their peers.

The mean scores of performing and witnessing dishonest behaviors were 0.45 ± 0.37 and 0.78 ± 0.47, respectively (on a 0–2 scale). Moreover, the mean score of perceived severity of dishonest behavior as being unethical was 3.34 ± 0.54 (on a 1–4 scale).

The most commonly performed and witnessed dishonest behaviors in the last semester were, respectively, “discussing about patients in public places or with nonmedical personnel” and “recording or reporting vital signs that had not been taken or recalled accurately” (items 1 and 2). The least commonly performed and witnessed behavior was “giving the wrong drug without reporting it” (item 11), (Table 1, pp. 15–17).

**Table 1:** The participants’ engagement and exposure to the dishonest behaviors in the clinical setting

Dishonesty behaviors in the clinical setting		Frequency of doing dishonest behavior in the previous semester					Frequency of witnessing clinical dishonest behaviors				
		never	once	twice or more	Missing	M (SD)	never	once	twice or more	Missing	M (SD)
1	Discussing patients in public places or with nonmedical personnel	171 (43.3)	107 (27.1)	117 (29.6)		0.86 (0.84)	98 (24.8)	133 (33.6)	163 (41.3)	1 (0.3)	1.16 (0.79)
2	Recording or reporting vital signs that are not taken or recalled accurately	214 (54.2)	105 (26.5)	75 (19)	1 (0.3)	0.64 (0.78)	139 (35.4)	108 (27.4)	146 (37)	2 (0.5)	1.01 (0.85)
3	Using uncertain data or fabricating patient information for assignments	270 (68.4)	83 (21)	42 (10.6)		0.42 (0.67)	182 (46.1)	123 (31.1)	90 (22.8)		0.76 (0.79)
4	Taking hospital supplies or medications from the hospital for personal use	281 (71.1)	82 (20.8)	31 (7.8)	1 (0.3)	0.36 (0.62)	198 (50.1)	95 (24.1)	102 (25.8)		0.75 (0.83)
5	Attempting to perform procedures on patients without adequate knowledge or failing to obtain guidance from instructors	245 (62)	110 (27.8)	40 (10.2)		0.48 (0.67)	162 (41)	123 (31.1)	110 (27.8)		0.86 (0.82)
6	Recording or reporting nursing care that is not performed	296 (74.9)	61 (15.4)	37 (9.4)	1 (0.3)	0.34 (0.64)	200 (50.6)	108 (27.3)	87 (22)		0.71 (0.80)
7	Not reporting incidents or errors involving patients	23 (59)	113 (28.6)	47 (11.9)	2 (0.5)	0.52 (0.69)	182 (46.1)	121 (30.6)	92 (23.3)		0.77 (0.80)
8	Breaking sterile techniques and neither reporting it nor replacing contaminated items	213 (53.9)	124 (31.4)	56 (14.2)	2 (0.5)	0.60 (0.72)	127 (32.1)	120 (30.4)	147 (37.2)	1 (0.3)	1.05 (0.83)
9	Recording patient responses to treatments or medications that are not assessed	290 (73.4)	72 (18.2)	31 (7.8)	2 (0.5)	0.34 (0.61)	217 (54.9)	116 (29.4)	61 (15.4)	1 (0.3)	0.60 (0.74)
10	Recording medications as administered when they are not	299 (75.6)	65 (16.5)	28 (7.1)	3 (0.8)	0.30 (0.59)	225 (57)	104 (26.2)	65 (16.5)	1 (0.3)	0.59 (0.75)
11	Giving the wrong drug without reporting it	314 (79.5)	55 (13.9)	21 (5.3)	5 (1.3)	0.24 (0.54)	232 (58.8)	110 (27.8)	51 (12.9)	2 (0.5)	0.53 (0.71)
12	Losing, breaking, or damaging patients’ belongings and not reporting it	286 (72.4)	81 (20.5)	28 (7.1)		0.34 (0.60)	229 (58)	116 (29.4)	50 (12.7)		0.54 (0.70)

The mean scores of perceived severity of dishonest behavior varied from 2.7 ± 0.92 in item 1 to 3.57 ± 0.76 in item 11. The most common behaviors considered “serious” by the participants were items 11 and 10, with a mean of 3.57 and 3.51, respectively. The lowest intensity was for items 1 and 4, with a mean of 2.7 and 3.19 respectively, (Table 2, pp. 18–19). Many participants had understood the perceived severity of clinical dishonesty as “no problem” or “minor problem”, which varied from 10.8% (item 11) to 35.4% (item 1).

**Table 2:** Participants’ responses about the ethical nature of academic dishonest behaviors in the clinical settings

Perceived severity of the unethical behavior		Unimportant	Least important	Important	Most important	Missing data	M (SD)
1	Discussing patients in public places or with nonmedical personnel	43 (10.9)	97 (24.5)	167 (42.3)	88 (22.3)		2.70 (0.92)
2	Recording or reporting vital signs that are not taken or recalled accurately	17 (4.3)	46 (11.6)	98 (24.8)	234 (59.3)		3.38 (0.85)
3	Using uncertain data or fabricating patient information for assignments	25 (6.3)	66 (16.7)	111 (28.1)	193 (48.9)		3.19 (0.93)
4	Taking hospital supplies or medications from the hospital for personal use	30 (7.6)	58 (14.7)	108 (27.3)	199 (50.4)		3.20 (0.95)
5	Attempting to perform procedures on patients without adequate knowledge or failing to obtain guidance from instructors	23 (5.8)	36 (9.1)	115 (29.1)	221 (56)		3.35 (0.87)
6	Recording or reporting nursing care that is not performed	13 (3.3)	34 (8.6)	1 (28.4)	236 (59.7)		3.40 (0.78)
7	Not reporting incidents or errors involving patients	9 (2.3)	38 (9.6)	128 (32.4)	220 (55.7)		3.41 (0.75)
8	Breaking sterile techniques and neither reporting it nor replacing contaminated items	12 (3)	34 (8.6)	85 (21.5)	264 (66.9)		3.20 (0.77)
9	Recording patient responses to treatments or medications that are not assessed	16 (4.1)	51 (12.9)	116 (29.3)	212 (53.7)		3.32 (0.85)
10	Recording medications as administered when they are not	17 (4.3)	29 (7.3)	81 (20.5)	268 (67.9)		3.51 (0.81)
11	Giving the wrong drug without reporting it	12 (3)	31 (7.8)	69 (17.5)	283 (71.7)		3.57 (0.76)
12	Losing, breaking, or damaging patients’ belongings and not reporting it	18 (4.6)	48 (12.1)	91 (23)	237 (60)	1 (0.3)	3.38 (0.87)

### Demographic variables related to clinical dishonesty

A significant difference was found in the mean score of clinical dishonesty in terms of gender, interest in the field of study, and the year of study (Table 3). The results of the follow-up test also showed a significant difference in the frequency of dishonest behavior between the participants who were and were not interested in nursing (*p* = 0.001). Furthermore, there was a significant difference between the group who were “somewhat interested in nursing” and those were “not interested” (*p* = 0.002). Moreover, significant differences were found between the second- and fourth-year students (*p* = 0.003) and the third- and fourth-year students (*p* = 0.001) in terms of the frequency of dishonest behavior committed by them.

**Table 3:** Prevalence of engagement in dishonest clinical behaviors according to demographic characteristics

Variables		N (%)	M (SD)	*P* value
gender	Male	194 (41.5)	0.50 (0.38)	0.006
	female	201 (58.5)	.41 (0.36)	
marriage	Single	337 (58.3)	0.46 (0.38)	0.630
	married	58 (14.7)	0.42 (0.33)	
Interested in nursing	Yes	182 (46)	.42 (0.35)	0.004
	Somewhat	177 (44.9)	.43 (0.35)	
	No	32 (8.1)	.71 (0.49)	
	missing	4 (1)		
Academic year	1st year	48 (12.1)	0.55 (0.42)	0.002
	2nd year	143 (25.3)	0.40 (0.31)	
	3rd year	104)26.3)	0.38 (0.36)	
	4th year	94 (23.8)	0.56 (0.40)	
	Missing	6 (1.5)		
Student work experience in a hospital	No	93 (23.5)	0.47 (0.36)	0.720
	Yes	298 (75.4)	.0.45 (0.38)	
	Missing	4 (1.1)		

Spearman correlation coefficient was used to investigate the relationship between performed dishonest behaviors with quantitative variables (age, GPA, witnessing dishonest behavior, perceived severity of dishonest behavior, and perceived clinical stress). The results showed that dishonest behaviors had a significant positive relationship with witnessing them (*r* = 0.53, *p* < 0.001), but a negative relationship with the perceived severity of behavior as an unethical behavior (*r* = − 0.40, *p* < 0.001), and a positive relationship with perceived clinical stress (*r* = 0.28, *p* < 0.001) (Table 4).

**Table 4:** Correlation between the prevalence of dishonest clinical behaviors and quantitative variables

	1	2	3	4	5	6
1-age	–	−0.065	0.093	0.201**^*^	0.054	0.064
2-GPA		–	−0.025	−0.055	0.024	0.321
3) Frequency engagement in dishonesty behavior in the last semester			–	0.53**	−0.405**	0.280**
4) Frequency observing the dishonesty behavior in the previous semester				–	−0.203**	0.310**
5) Perceived severity as being unethical					–	−0.223**
6) perceived clinical stress						–

* *P* value <0.05, ** *P* value <0.01, *** *P* value <0.001

## Discussion

This study showed that most participants (89.1%) had done at least one clinical dishonest behavior. This is more than the rate reported in a Korean (66%) [13] and an American (54%) [10] study. However, the questionnaire used in those studies had 10 items, while the questionnaire used in the present study had 12 items, which could increase the frequency of reporting dishonest clinical behaviors. In the study conducted by Schneider and McClung, 96% of nursing students were involved in at least one academic or clinical dishonest behavior [14].

The results of the present study showed that the most commonly performed dishonest clinical behaviors were the most commonly witnessed ones, which were “discussing about patients in public places or with nonmedical personnel” and “recording or reporting vital signs that had not been taken or recalled accurately”, respectively. According to Bandura’s social learning theory [19], observing the behavior of others (peers in this study) can contribute to engaging in that behavior.

The least frequently reported dishonest behavior was “giving the wrong drug without reporting it”. Moreover, the results revealed that the mean score of students’ perceptions of dishonest behavior related to drug therapy (items 10 and 11) was higher than other items. Furthermore, it was found that the students were more likely to be sensitive to behaviors that have a direct impact on patients’ health and did not engage in such unethical behaviors due to fear of adverse health consequences for the patient and educational and legal consequences for themselves. However, they did not think talking about the patient’s conditions in public places and recording inaccurate vital signs could be dangerous to the patient or could be considered a sign of immorality.

Moreover, the results showed that the mean frequency of clinical dishonesty was significantly lower in females. This result is consistent with the findings of Krueger [10] and Amini et al. [20] but contradicts the findings of Park et al. [13].

According to the results, the frequency of clinical dishonesty was related to being interested in nursing; thus, the frequency of these behaviors in students who were “interested” and “somewhat interested” was less than that of those who were “uninterested”. Also, a study found that students who were satisfied with nursing were likely to have higher ethical awareness, be more faithful to their discipline and its ethics, and exhibit less dishonest behaviors [21]. In Iran, most students typically choose their field of study based on their scores on the Iranian University Entrance Exam (IUEE). Thus, sometimes they just want to become a university student, regardless of their interest, or they select nursing for its relatively good job market. Considering the importance of the nursing profession and its impact on the health of the community, it is recommended that nursing students be interviewed before admission to nursing programs to ensure that they are interested in their field of study and to prevent the entry of uninterested persons into nursing schools.

Besides, according to our findings, the highest frequency of clinical dishonesty was observed among fourth-year students, which could be related to their more frequent presence in the clinical setting, more workload, and less supervision by instructors. In the Iranian Nursing Curriculum, only senior students attend internships, do not have to pass theoretical courses, and are in the clinical setting almost 7 days a week. Krueger found that an increase in clinical work time was also associated with an increase in clinical dishonesty [10]. Park et al. also reported more dishonest clinical behaviors among senior students [13].

According to the results of the present study, the age and GPA of students were not correlated with the frequency of their dishonest behaviors, which is in line with other studies [10, 13, 22]. Moreover, the results showed that witnessing clinical dishonesty had a significant positive relationship with dishonest clinical performance. That is, the more people witness their coworkers’ unethical behavior, the more they are likely to do it. This result is in line with Bandura’s social learning theory.

The results of this study indicated that the frequency of dishonest behavior was inversely correlated with its perceived severity as unethical behavior, indicating that nursing students’ behaviors in the clinical setting are influenced by their beliefs and faiths. This can be promising for nursing educators and administrators because it seems that educating students about professional ethics can reduce their dishonest and unethical behaviors. Park et al. also found that perceived severity of fraudulent behaviors was predictive of classroom cheating, but it was not associated with clinical dishonesty [13].

The results of the present study demonstrated that the higher the perceived stress score in the clinical setting, the greater the frequency of clinical dishonesty. Nursing students may use academic dishonesty as a coping strategy to reduce stress [23]. People who engage in these behaviors are not morally troubled, but external factors and pressures, rather than the intrinsic nature of the individual, are the main reasons behind their dishonest behavior. Factors such as fear of failure, inefficiencies in clinical practice, competition with peers, and excessive expectations of educators lead to stress in nursing students [23–25], which, in turn, can be a source of dishonest behavior. Since negative coping strategies lead to increased stress, nursing students should be educated on effective coping strategies to effectively deal with clinical stresses.

## Conclusion

The results of this study showed that dishonest clinical behaviors committed by nursing students were serious to the extent that they could be problematic. Clinical dishonesty may become a recurring pattern of professional behavior and affect patient health and safety. Thus, first, nursing managers and educators should select those students who are interested in the nursing profession. Then, they should provide necessary training to the students regarding the impact of ethical behaviors on patient care. In addition, they should teach the students on how to use effective strategies to cope with clinical stresses to ensure patients’ health. Therefore, interventional studies should be conducted to investigate the impact of reducing students’ clinical stress on their dishonest performance. Since students who show clinical dishonesty may continue to perform such behaviors as nurses in the future, clinical nursing managers need to be sensitive to the issue of clinical dishonesty.

### Limitations

The limitations of this study were data collection using questionnaires, using a cross-sectional design, and employing a census sampling method. More studies need to be conducted to explore the relationship between clinical stress and clinical dishonest behaviors in different settings and also to understand the causes of clinical dishonest behaviors among nursing students.

## Supplementary information

**Additional file 1.** Questionnaire 1: Clinical Dishonesty Questionnaire

**Additional file 2.** Questionnaire 2: Nursing student perceived clinical stress scale (NSPCSS)

## Data Availability

The datasets used and/or analyzed during the current study are available from the corresponding authors on reasonable request.

## Abbreviations

GPA: Grade point average
MSc: Master of Science
