# Supplementary material for: The relationship between clinical dishonesty and perceived clinical stress among nursing students in southeast of Iran
Source: BMC Nurs. 2020 May 14;19:39. doi: 10.1186/s12912-020-00434-w (PMC7227112; doi:10.1186/s12912-020-00434-w)
Supplement: Supplementary file 1 — Additional file 1. Questionnaire 1: Clinical Dishonesty Questionnaire [file 12912_2020_434_MOESM1_ESM.docx]

**Clinical Dishonesty Questionnaire**

| Dishonesty behaviors in the clinical setting | | Frequency of doing dishonest behavior in the previous semester | | | Frequency of witnessing clinical dishonest behaviors | | | perceived severity of the unethical behavior | | | |
| --- | --- | --- | --- | --- | --- | --- | --- | --- | --- | --- | --- |
|  |  | never | once | twice or more | never | once | twice or more | unimportant | least important | important | most important |
| 1 | Discussing patients in public places or with nonmedical personnel |  |  |  |  |  |  |  |  |  |  |
| 2 | Recording or reporting vital signs that are not taken or recalled accurately |  |  |  |  |  |  |  |  |  |  |
| 3 | Using uncertain data or fabricating patient information for assignments |  |  |  |  |  |  |  |  |  |  |
| 4 | Taking hospital supplies or medications from the hospital for personal use |  |  |  |  |  |  |  |  |  |  |
| 5 | Attempting to perform procedures on patients without adequate knowledge or failing to obtain guidance from instructors |  |  |  |  |  |  |  |  |  |  |
| 6 | Recording or reporting nursing care that is not performed |  |  |  |  |  |  |  |  |  |  |
| 7 | Not reporting incidents or errors involving patients |  |  |  |  |  |  |  |  |  |  |
| 8 | Breaking sterile techniques and neither reporting it nor replacing contaminated items |  |  |  |  |  |  |  |  |  |  |
| 9 | Recording patient responses to treatments or medications that are not assessed |  |  |  |  |  |  |  |  |  |  |
| 10 | Recording medications as administered when they are not |  |  |  |  |  |  |  |  |  |  |
| 11 | Giving the wrong drug without reporting it |  |  |  |  |  |  |  |  |  |  |
| 12 | Losing, breaking, or damaging patients’ belongings and not reporting it |  |  |  |  |  |  |  |  |  |  |
