# Supplementary material for: The relationship between clinical dishonesty and perceived clinical stress among nursing students in southeast of Iran
Source: BMC Nurs. 2020 May 14;19:39. doi: 10.1186/s12912-020-00434-w (PMC7227112; doi:10.1186/s12912-020-00434-w)
Supplement: Supplementary file 2 — Additional file 2. Questionnaire 2: Nursing student perceived clinical stress scale (NSPCSS) [file 12912_2020_434_MOESM2_ESM.docx]

**Nursing student perceived clinical stress scale (NSPCSS)**

| never | rarely | sometimes | often | always | **Inadequate knowledge and skills** | **A** |
| --- | --- | --- | --- | --- | --- | --- |
|  |  |  |  |  | Student’s inadequate knowledge for patient care | 1 |
|  |  |  |  |  | Student’s inadequate experience in patient care | 2 |
|  |  |  |  |  | Student’s inadequate skills for patient care and equipment use | 3 |
|  |  |  |  |  | **Instructors limited clinical competence** | B |
|  |  |  |  |  | Instructor’s inadequate attention and guidance | 4 |
|  |  |  |  |  | Difference between instructor’s education and student’s educational needs | 5 |
|  |  |  |  |  | Instructor’s limited skills | 6 |
|  |  |  |  |  | Instructor’s use of traditional teaching methods and routine in clinical education | 7 |
|  |  |  |  |  | Instructor failure to provide independence for students | 8 |
|  |  |  |  |  | Over emphasis of theoretical training (as opposed to applied clinical education by instructor) | 9 |
|  |  |  |  |  | **Inappropriate clinical environment** | C |
|  |  |  |  |  | Inadequate equipment for appropriate nursing care | 10 |
|  |  |  |  |  | Shortage of recreational and educational facilities in the clinical environment | 11 |
|  |  |  |  |  | Observing the violation of patient rights by healthcare providers | 12 |
|  |  |  |  |  | Students exploitation by healthcare providers | 13 |
|  |  |  |  |  | Observing non-standard care delivery to a patient by others | 14 |
|  |  |  |  |  | Inadequate time for appropriate nursing care | 15 |
|  |  |  |  |  | Fatigue due to heavy physical workload | 16 |
|  |  |  |  |  | Receiving inadequate support from healthcare providers | 17 |
|  |  |  |  |  | Misconduct by a patient or family member | 18 |
|  |  |  |  |  | Inconsistency between the theoretical and clinical education explanation provided. | 19 |
|  |  |  |  |  | **Inefficient clinical education planning** | ِD |
|  |  |  |  |  | Vague job description | 20 |
|  |  |  |  |  | Vague explanations of the objectives of clinical education | 21 |
|  |  |  |  |  | Instructors’ personalized approach to the use of educational rules and regulations | 22 |
|  |  |  |  |  | Inappropriate planning for clinical education by school authorities | 23 |
|  |  |  |  |  | **Instructor’s inappropriate conduct** | E |
|  |  |  |  |  | Instructor’s inappropriate conduct in the case of student error | 24 |
|  |  |  |  |  | Instructor’s high expectations | 25 |
|  |  |  |  |  | Instructor’s unfair evaluation | 26 |
|  |  |  |  |  | Lack of instructor’s feedback after doing a task | 27 |
|  |  |  |  |  | Instructor’s insufficient education about personal safety | 28 |
|  |  |  |  |  | Feeling of bafflement due to contradiction by some instructors | 29 |
|  |  |  |  |  | **Concerns over the characteristics of nursing** | F |
|  |  |  |  |  | Concern over affliction of psychological problems during patient care | 30 |
|  |  |  |  |  | Concern over legal problems due to negligence or error in patient care | 31 |
|  |  |  |  |  | Concern over affliction of physical problems during patient care | 32 |
